# Surface Modifications by Field Induced Diffusion

**DOI:** 10.1371/journal.pone.0030106

**Published:** 2012-01-13

**Authors:** Martin Olsen, Magnus Hummelgård, Håkan Olin

**Affiliations:** Department of Natural Sciences, Engineering and Mathematics, Mid Sweden University, Sundsvall, Sweden; Joint Research Centre - European Commission, Germany

## Abstract

By applying a voltage pulse to a scanning tunneling microscope tip the surface under the tip will be modified. We have in this paper taken a closer look at the model of electric field induced surface diffusion of adatoms including the van der Waals force as a contribution in formations of a mound on a surface. The dipole moment of an adatom is the sum of the surface induced dipole moment (which is constant) and the dipole moment due to electric field polarisation which depends on the strength and polarity of the electric field. The electric field is analytically modelled by a point charge over an infinite conducting flat surface. From this we calculate the force that cause adatoms to migrate. The calculated force is small for voltage used, typical 1 pN, but due to thermal vibration adatoms are hopping on the surface and even a small net force can be significant in the drift of adatoms. In this way we obtain a novel formula for a polarity dependent threshold voltage for mound formation on the surface for positive tip. Knowing the voltage of the pulse we then can calculate the radius of the formed mound. A threshold electric field for mound formation of about 2 V/nm is calculated. In addition, we found that van der Waals force is of importance for shorter distances and its contribution to the radial force on the adatoms has to be considered for distances smaller than 1.5 nm for commonly used voltages.

## Introduction

A voltage pulse between a scanning tunneling microscope (STM) tip and a surface will modify the surface under the tip creating either a mound or a pit on the surface, see ref. [1], [2] and Table 1.

**Table 1:** Mound or pit formation for different tip and surface materials and tip polarity.

Reference	Tip	Surface	Effect of negative tip	Effect of positive tip
Mamin (1990) [3]	Au	Au	Mound	Mound
Hsiao (1994) [5]	Au or Cu	Si	Au or Cu mound^1^	Non-metallic mound^1^
Bessho (1994) [9]	PtIr	Au	Pit	Mound
Mascher (1994) [8]	Au	Au	Pit^2^	Mound^2^
Chang (1995) [24]	Au, W, PtIr	Au	Mound, Crater^3^	Mound, Crater^3^
Kondo (1995) [17]	W or Pt	Various^4^	Pit	-
Ohi (1995) [12]	Au	Au	Pit, Mound^5^	Mound
Hu (1998) [7]	Al	Si	Mound	Mound
Mayer (1999) [14]	W	Au	Mound	-
Zhang (2001) [6]	Au	Au	Mound, Crater	Mound, Crater
Park (2002) [10]	Al	Si	Erasure of mound	Increased mound size
Park (2002) [10]	Au	Si	Incresed mound size	Erasure of mound
Fujita (2003) [11]	Ag	Si	Mound	Mound

1 : Element determination by scanning Auger microprobe spectra.

2 : For triangular voltage pulse. Rectangular pulse used elsewhere.

3 : Nonconducting liquid between tip and surface.

4 : Au, Ag, In, Si, Pt, W, C, SiO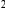, MoS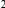 or Bi
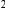
Sr
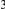
CaCu
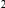
O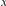.

5 : Pit created at larger tip-surface distance, mound at smaller.

The surface atoms on the tip or sample are subjected to an electrostatic force and this can lead to two different scenarios: either field evaporation [3]–[12] or field enhanced diffusion [4], [13]–[16] of atoms. However, field evaporation as Tsong [4] and others [17] has pointed out, is less likely compared to diffusion for the low voltages used in the experiments. In field evaporation an electric field of 20–50 V/nm is required [18] while typical voltages use in these experiments are 1–5 V/nm. In field enhanced diffusion, surface atoms (adatoms) are hopping between equivalent equilibrium positions due to lattice vibrations [19], [20], and if they posses dipole moments they may be attracted towards the tip by the inhomogeneous electric field between the tip and the surface making a mound.

At tunneling distances the van der Waals force is also of importance because it grows as the inverse fourth power of distance [21], see equation (5). However, this contribution has been little discussed in the literature [22]. The situation is complicated by the proximity between tip and sample, which is only about one nanometer when tunneling occur, that might lead to the formation of a neck between tip and sample [6], [23], [24]. At larger distances, however, we can neglect the neck formation mechanism [12].

The experimental results, summarized in Table 1, seems to be somewhat contradictory but can be understandable if considering the complexity of the system: different set of parameters make different mechanisms dominate. We have several parameters to take into consideration: 1) tip-to-surface distance, 2) tip radius, 3) applied voltage polarity and magnitude and 4) material in the tip and sample.

**Figure 1:**
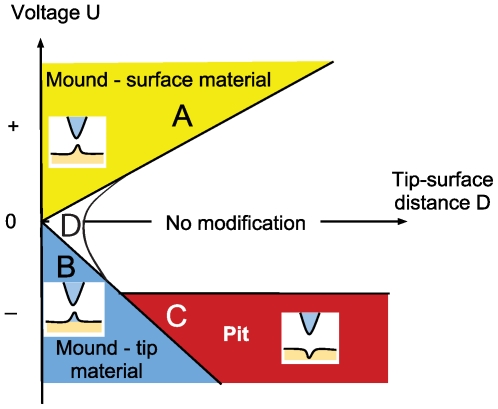
Phase diagram showing STM induced surface modifications at different tip voltage 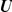 and different tip-to-surface distance 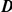 mainly constructed using ref. [5], [12] and ref. [17]. In drawing the lines for mound formation we have assumed that it is the electric field 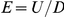 that decides if some particular kind of mound will be formed. Mounds will form at positive voltages, area A. From our model assuming field induced diffusion of adatoms we can calculate the threshold electric field for mound formation for area A to 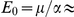
2 V/nm. In area B we have transfer of tip materials to the surface making a mound of tip material on the surface. For pits, area C, we have assumed that they are formed at constant 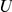 independent of the tip-to-surface distance 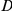 in agreement with Kondo *et al.* [17]. At short distances and low electric fields, area D, the van der Waals force will contribute in creating a mound [22]. Close to the U-axis (not shown) at electrical fields above 20–50 V/nm field evaporation will occur [18].

The *phase diagram* in fig. 1 is a schematic of the formation of mounds and pits using the tip-surface distance and the voltage as parameters. It is mainly constructed using ref. [5], [12] and ref. [17]. For positive tip we get mounds, area A in the phase diagram of fig. 1. For negative tip mounds form at shorter distances, area B, while pits are formed at larger distances, area C. This diagram is consistent with the references in Table 1, except for ref. [10]. However, the possibility of transport of positive as well as negative ions may likely explain this disagreement; negative ions are transported from negative to positive electrode and vice versa [4].

In area A with positive tip, we obtain mounds, made up of sample material likely created by field enhanced surface diffusion of adatoms. The mounds created using positive tip are unstable lasting only an hour in the experiment of ref. [5]. Mayer *et al.* [14] made a computer simulation of such a field enhanced diffusion of adatoms under an STM tip. From our model (see below) on electric field induced diffusion we can calculate the threshold electric field for mound formation for area A: inserting equation (25) for the threshold voltage 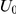 into equation (18) for the electric field 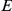 yields for 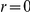 the threshold field for mound formation 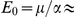
2 V/nm.

In area B, at short distances and negative tip voltage, mounds made of tip materials are formed as found by Hsiao et al [5]. The transport of tip material is due to field enhanced diffusion on the tip toward the gap, leading to a neck formation, that result in a mound when retracting the tip [5]. If the applied voltages is increased high enough, field evaporation of tip material will instead occur [5].

In area C, the experiments by Kondo *et al.* [17], [25] show a strong correlation between the threshold voltage for pit formation and the binding energy for ten different materials: for example Au with a binding energy of 3.8 eV has a threshold voltage of 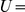
3.5 V, while W with binding energy of 8.8 eV has 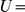
8.7 V. Their explanation for this mechanism is sublimation induced by tunneling electrons.

In area D, for short distances and low electric fields, Erts *et al.* [22] measured the force between a gold coated atomic force microscope (AFM) tip and a gold tip using an AFM cantilever placed inside a transmission electron microscope (TEM). They could in this way image the system while manipulating it. They found an anomalous high value of the jump-to-contact distance indicating a larger force than expected from the distances using the van der Waals force. Closer inspection revealed a thin neck formed between the tip and sample. Their interpretation was that the van der Waals force caused field induced surface diffusion leading to shorter gap which in turn increased the van der Waals force, and this avalanche of adatoms quickly formed the neck.

In this paper, we describe a field enhanced surface diffusion model (area A) and also a model at short distances and low electric fields where van der Waals forces are contributing (area D).

We develop a simple analytical model to calculate the static electric forces from the tip governing the motion of the adatoms. We extend the model of Mayer *et al.* [14] by including the surface induced dipole moment which introduces a force on the adatoms that depend on the polarity of the applied voltage between the tip and the surface. We find a novel formula for the threshold voltage for mound formation for positive tip as well as a relation between the mound radius and the applied voltage. In addition we include the van der Waals force described in ref [21], [22]. We find that its contribution to the radial force on the adatoms is small for distances larger than 1.5 nm for commonly used voltages.

## Analysis

### Van der Waals force

In this section we will calculate the van der Waals interaction energy of an adatom with a parabolic tip, representing an STM tip, and thereafter the force. The (non retarded) van der Waals interaction energy between two atoms at distance 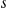 from each other is given by [21], [26]

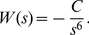
(1)


**Figure 2:**
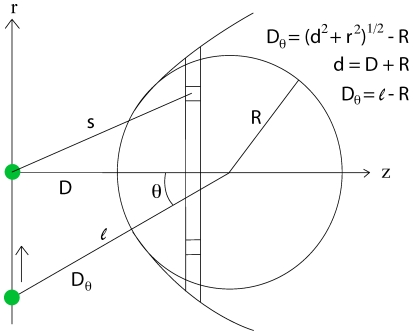
Calculation of van der Waals force on an adatom by a massive paraboloid tip with radius of curvature 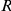 at the apex. For a adatom sitting off-axis on the surface the distance to the tip is 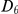. The letters denoting distances, 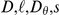 and 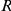 are placed at the midpoint of the distances they represent.

An approximate value of the constant 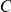 for identical atoms is given by 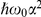, see ref. [26], where 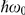 is the energy of the strongest optical absorption line and 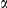 is the polarizability of the atoms. Following Israelachvili [21] we have for a ring-shaped element in the tip around the z-axis at radius 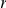 the volume 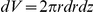, see fig. 2. The number of atoms in the ring is then 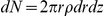 where 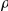 is the number density of the tip material. The interaction energy between the ring and an adatom on the surface at 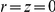 is then
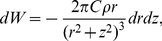
(2)using 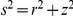. If the ring is a segment in a parabolic tip given by 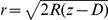 with apex at 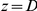 and extending into infinity, the interaction energy for the adatom at 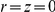 with the tip is
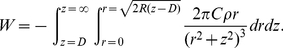
(3)Integrating we obtain
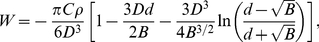
(4)where 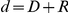 and 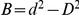.

A plot of 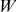 as a function of 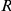 shows that it varies monotonically from 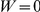 at 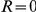 to 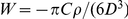 as 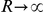. The force *on the adatom* right under the tip is then 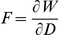, so that the force is directed towards the tip. Using this equation (4) yields
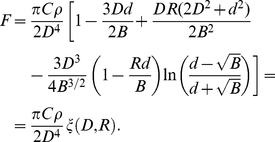
(5)This equation can be used to calculate the van der Waals force on a adatom *right under the tip*. Plotting 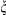 in equation (5) as a function of 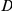 shows that it decreases monotonically from 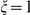 at 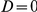 towards zero as 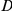 approaches infinity. A plot of 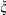 as a function of 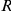 yields 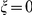 at 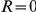 and then increases monotonically towards 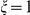 as R approaches infinity.

We now consider the van der Waals forces on the adatom when it is located on a conducting surface under the tip. The van der Waals force on an adatom *by the surface* is always perpendicular to the surface balancing the z-component of the force from the tip. To see how the adatom is moving it is thus more interesting to study the r-component. If we have the force 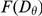 directed towards the center of the sphere approximating the parabolic tip, the force components on an adatom sitting off-axis on the surface are
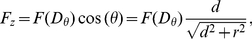
(6)

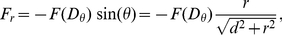
(7)where 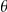 is the off-axis angle counted from the center of the sphere, see fig. 2. 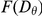 is obtained from equation (5) by replacing 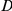 with the expression 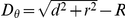. This is the radial distance 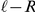 from the tip to the surface for an off-axis angle if the tip is approximately considered spherical seen from a point on the surface.

Approximating with 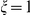 in (5) we obtain a simpler formula which yields a *upper limit* of the van der Waals force. The radial component becomes
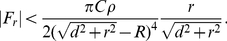
(8)


### Electrostatic dipole force

**Figure 3:**
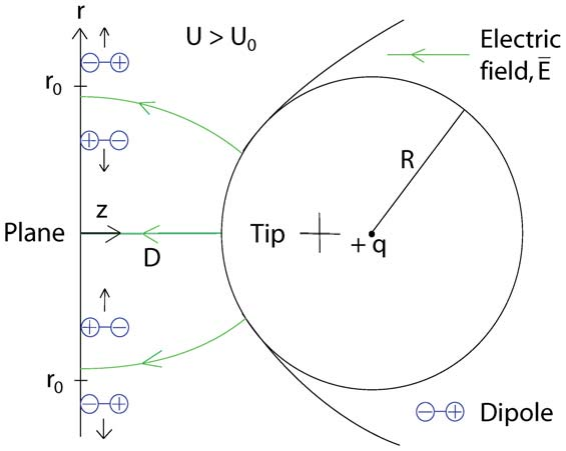
Adatoms with dipole moments on the surface for 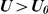. The dipole moment 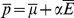 are tending to align to the strong field inside 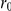 on the surface and points away from the surface outside 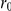 where the field is weaker, see equation (20). The radial force on the dipole on the surface is due to this effect attractive inside 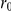 and repulsive outside, tending to create a mound made of surface material with maximum radius 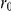.

In this section we will calculate the static electric dipole force on an adatom when we have a voltage applied between the tip and the surface. To obtain a simple model we approximate the field as given by a point charge 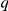 located at a distance 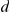 over an infinite flat conducting surface. The midpoint of the adatom is located a distance 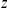 over the surface, see fig. 3. The potential 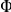 at a point is then given by
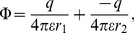
(9)where we have 
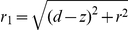
 and 
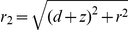
. The first term is the potential due to the charge 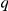 and the second term is the potential due to the mirror of the charge 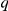 in the conducting surface. 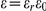 is the permitivity of the medium between the charge and the surface. To obtain a useful model we must find an expression for the charge 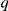 as a function of the applied voltage 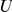 between the tip and the conducting surface. On the line connecting the charge 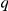 and its mirror 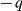 (i.e. 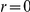) we have at the distance 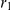 from 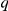:
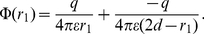
(10)


We see from equation (10) that if 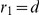, that is we are in the surface, we have 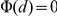. The voltage between the tip apex, at a distance 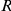 from 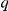, and the surface is then given by 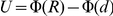. So
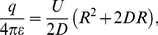
(11)using 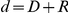.

We here assume as a model that the equivalent charge 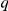 is fixed at the center of curvature of the tip independent of the tip-to-surface distance 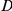. This is only approximately true but leads to equation (18) that is true both in the limit of 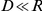 where we obtain 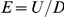 and in the limit 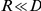 where we obtain 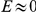.

We can now calculate 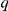 if we know 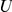, 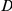 and 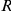. 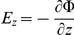 and 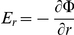 yields
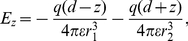
(12)

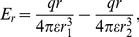
(13)

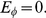
(14)It is interesting to study the field close to the surface, that is when 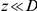 (implying 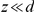). To do so we expand equation (12) and (13) in Taylor series around 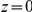. We then obtain
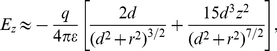
(15)

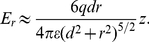
(16)At 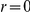 we have 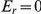 because on the surface right under the charge by symmetry the only field component that exists is 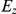. 
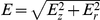
 . Dividing equation (16) with (15) we obtain
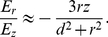
(17)Because 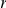 may be of about the same order as 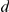 and because we in the Taylor expansion have assumed that 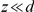 this quota is small compared to unity. Thus we may neglect 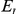 compared with 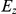 when calculating 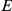. Using equation (11) in equation (15) for the z-component of the electrostatic field we then obtain in the surface (where 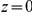) using 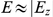:
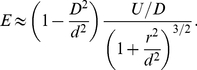
(18)The dipole moment 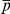 for an adatom in an electric field 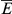 is given by Tsong and Kellogg [20]:
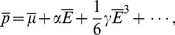
(19)where 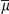 is the surface-induced dipole moment of the adatom, 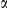 is its polarizability and 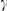 is its hyperpolarizability. 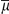 always points away from the surface that it sits on, see ref. [27], [28], so 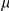 is always positive in our calculations regardless of the polarity of the voltage 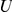. We have for the 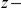 and 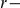
components of the dipole moment 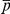, neglecting the hyperpolarizability term:
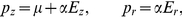
(20)where 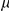 is positive. 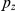 and 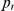 may therefore be positive or negative. The force on a dipole in an electric field is
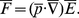
(21)Using equation (11), (20) and (15)–(16) and their derivatives into equation (21) we obtain to order 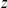


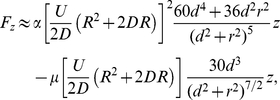
(22)

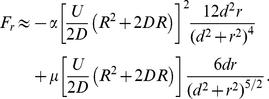
(23)where 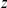 is the height over the surface for the center of the adatom, about one adatom radius. The distance 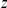 are only affecting 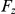, not 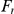, as we see from equations (22) and (23). We are now able to calculate the electrostatic forces on the adatom on the surface under the tip. The calculation above is valid for the first step in the mound formation where the surface still may be considered as flat. Mayer *et al.* [14] use 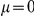 in their model so they do not obtain the polarity dependence.

## Results and Discussion

To calculate the force we need to estimate the surface-induced dipole moment and the polarizability of an adatom. For metal atoms we have 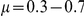 Debye and 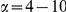 Å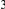, see ref. [20]. We use the average values 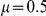 Debye and 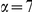 Å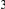. Using SI-units this is 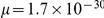 Cm and 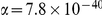 Cm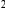/V. We assume that the adatom is a gold atom with a center height over the surface of about an atomic radius of gold so 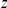 = 0.14 nm. Atoms are detaching from steps on the substrate and becomes adatoms [14]. In this paper we only assume that the adatoms are on the surface and calculate how they move.

**Figure 4:**
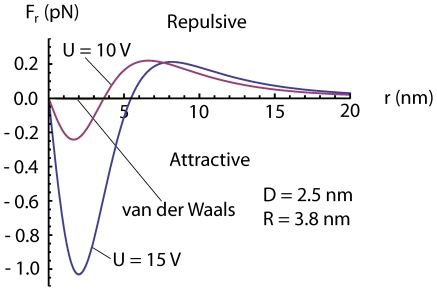
Radial dipole force on an adatom obtained from equation (23)using 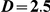 nm and 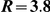 nm and our values of 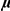 and 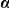 for two different voltages 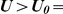
6.5 V. Increasing all the distances 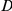, 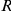 and 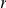 and the voltage 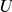 by a factor of 10 yields a reduction of the force 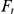 to 1/10. The radial van der Waals force calculated using equation (7) is barely visible in the figure at this “large” 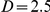 nm.

**Figure 5:**
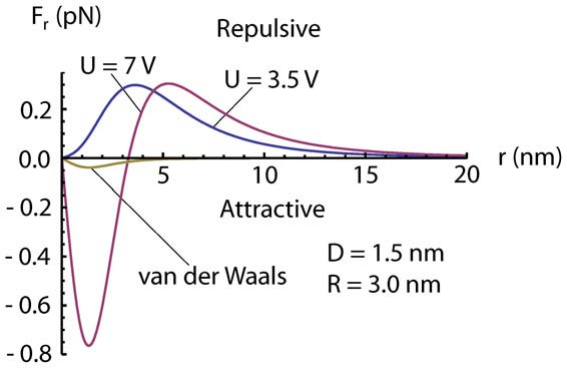
Radial dipole force on an adatom obtained from equation (23) using 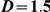 nm and 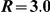 nm and our values of 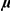 and 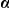 for two different voltages 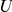, one above and one below 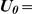
3.7 V. The van der Waals force has been calculated using equation (7) and plotted in the figure using 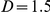 nm. For 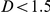 nm the van der Waals force becomes important for the radial force on the adatoms.

**Figure 6:**
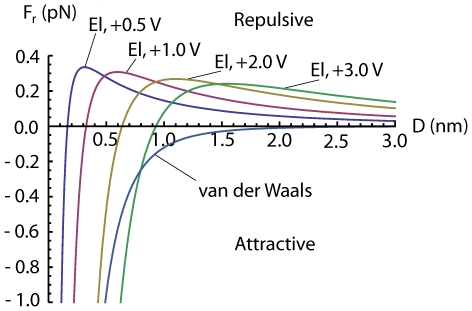
Radial van der Waals force from equation (7) and radial electric forces (El) from equation (23) for different tip voltages, at 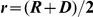 as a function of tip-to-surface distance 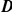. 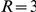
 nm.

If a dipole in a inhomogeneous electric field is free to rotate or is created due to polarization by an electric field, the dipole is always attracted towards stronger field i.e 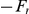 is positive. But the component of the dipole moment which is due to surface induction 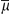 always *points away* from the surface[27], [28], making the electric force in equation (23) repulsive at some voltages and distances. This means that we may have an attractive force from the center under the tip out to some equilibrium radius where the force change sign and becomes repulsive. The force calculated from (23) is small for the commonly used voltage and distances, of order of 1 pN, see fig. 4, fig. 5 and fig. 6, but because the adatoms are hopping on the surface due to thermal vibration even a small net force can be significant in the drift of adatoms [19], [20]. Setting 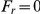 in equation (23) then yields
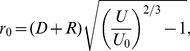
(24)where
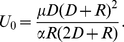
(25)


Using for example 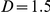 nm and 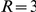 nm we obtain 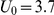 V. 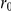 is the equilibrium radius on the surface at voltage 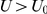 where the force changes sign from attractive to repulsive as we are moving out from the center right under the tip towards infinity, see fig. 3. For voltages 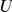 between zero and 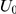 the electrostatic force is repulsive for all distances 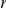, that is there is no equilibrium distance.

Inserting equation (25) for the threshold voltage 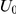 into equation (18) for the electric field 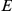 yields for 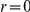 (a small mound with radius zero is barely formed according to the definition of threshold voltage, equation (24)) the threshold field for mound formation 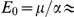
2 V/nm.

We have thus two novel findings for positive tip voltages: a threshold field for mound formation of about 2 V/nm and a voltage dependent radius of the mound, equation (24).

For negative voltages, in contrast to positive voltages, the force 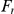 is attractive for all distances, except at 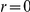 where the force is zero, as we see from equation (23), so negative voltage is tending to creating a mound on the surface with no threshold voltage. This due to that in this case the dipole moment 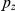 does not change sign at a certain field as we have at positive tip.

The experimental findings are somewhat contradictory: a more or less sign independent threshold voltage of about 3.5 V is observed according to ref. [3], [24]. However, mound formation is less reproducible with positive voltage at the tip [11], [24]. A mound created by a tip-positive pulse could be removed by applying a negative pulse in some cases [9]. Using a gold (copper) tip over a silicon surface, a negative voltage on the tip yields a gold (copper) mound on the surface, but for positive voltage the mound are not made up of metal [5]. This indicates that at negative tip, materials from the tip are transferred to the surface while at positive tip surface material are attracted towards the area under the tip creating a mound. Using a gold tip and a gold surface, at high tunneling resistance (large tip to surface distance) at a negative tip a hole was created and at positive tip a mound was created [12].

However, at low tunneling resistance (small tip to surface distance) a mound adjacent to a pit was often created by a negative tip where the current kept flowing even after the voltage pulse, indicating necking between surface and tip [12]. Transfer of material between tip and surface seems to be possible both ways depending on the materials used, see ref. [10] and table 1.

Mayer *et al.* [14] simulated the diffusion of adatoms on a surface using 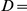
25 nm, 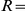
38 nm and 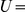
−100 V at the tip. (They also measured experimentally the size of a mound while it was growing under the tip.) They obtained a mound diameter (at which the growth rate is zero) of 40 nm. Their simulation model was independent of the sign of the voltage so 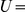
+100 V would have given the same result. To compare this with our model we calculate the threshold voltage using equation (25) in our paper and the 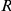 and 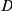 in the simulation using our estimate 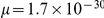 Cm and 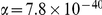 Cm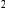/V for a metal adatom. We then obtain 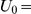
65 V. Thereafter we calculate the equilibrium radius at 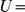
100 V using (24). We then obtain a radius of 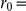
36 nm in good agreement with the simulation result. However, this should be a coincidence because the mechanisms are different in the two cases.


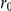
 is the maximum radius of the mound in our model because no adatoms can be attracted from outside of this radius. In the simulation made by Mayer *et al.* adatoms are attracted from all radius making the mound thickness grow inside some radius etching away surface material outside this radius. The similar results though increases the reasonability of our approach to the field induced diffusion model.

Calculating the van der Waals force, we find that it is relatively small compared to the dipole force for distances larger than 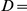
1.5 nm using voltages commonly used in experiments. However, at small distances the van der Waals force will dominate over the electrostatic dipole force [22], [29], see fig. 4, fig. 5 and fig. 6. For the van der Waals force we have 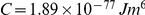, see ref. [21], and the number density of the material in the tip is estimated as 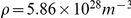 yielding the value of the factor 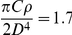 pN for 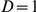 nm in equation (5). The radial component on an off-axis adatom will be smaller than this according to equation (7). An upper limit for the radial van der Waals force is given by equation (8). Van der Waals induced surface diffusion dominates for this small distances for commonly used voltages. This effect may explain the neck formation obtained by Erts *et al.*, [22], see text for area D in the introduction. The effect of adding the radial van der Waals force to the dipole force is that it tends to lower the threshold voltage for mound formation at positive tip making the mounds larger at a given applied voltage.

Other effects that may influence the mound formation is charge disorder effects, dipole-dipole interaction and dislocation activity which we will discuss below. Charge disorder interactions, where we have fluctuation-induced interaction between randomly charged dielectrics, is considered by ref. [30], [31]. At larger distances this effect can dominate over the van der Waals force. How does this affect the mound formation? A length scale for the total force 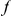 as a function of the distance 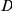 is the Bjerrum length, 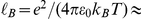
56.8 nm at room temperature, see [31]. We see from fig. 2 in [31] that for our distances 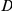, about 1–50 nm, we have for quenched charge disorder 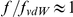 that is, the van der Waals force is dominating. For annealed charge disorder they obtain 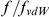 between 1 and 1.6 for all distances so even here is the total force of the same order as of the van der Waals force. Thus for our distances charge disorder effects can be neglected when the van der Waals force can be neglected. The dipole-dipole interaction between adatoms on the surface should be less important because their interaction should seem to average out. This due to that the adatoms position on the surface are random so their average behaviour would mainly be determined by the field from the tip. Changes in temperature should only effect the diffusion rate [19] but not the equilibrium radius 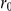 because it does not affect the force 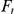 which is given by the electric field. Recently, Mordehai *et al.* [32] finds, using a molecular dynamics simulation, that jump-to-contact between nanoparticles likely is due to dislocation activity. The particles are initially dislocation free and are in this state even after the jump-to-contact, they call this pseudoelasticity. This process is according to the simulation faster than competing processes like surface diffusion and may play a role in the formation of a mound in area D in fig. 1.

To test our model, one way would be to measure the mound radius as a function of applied voltage for tip-positive voltage pulse. In the literature we have only seen this experiment done for a negative tip, see Fujita *et al.* [11]. The lacking of experimental data for positive tip might be due to the experimental difficulty to reproduce mound formation for this tip polarity [11], [24].

If one wants to create mounds on a surface, using negative tip is probably more effective. The mounds seems then be made up by tip material which may make the mounds more stable [5] than mounds constructed of surface material using a positive tip. However, for dynamic use in electronics we may not want the mound to be stable. Because the size of the mound for positive tip is limited by 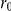 the mound formation may be more controlled in this case than at negative tip where we may have unrestricted growth of the mound trying to bridge the gap between tip and surface.

The same type of behaviour that we see on the surface under a STM-tip we may have on the tip itself, with mound formation above a threshold voltage. If we have two electrodes of different shape (tip radius) and material the threshold voltage for mound formation may be different on the two electrodes. By choosing a material with low mobility of adatoms as one electrode we may prevent mound formation on this electrode.

A way to monitor most of the parameters directly is to do the experiment using an STM inside a transmission electron microscope (TEM) where one can image the tip-surface system while manipulating it [33], [34]. All important parameters are accessible by TEM imaging: the tip-surface distance, tip radius, movement of tip or sample material, while the STM provide tip motion and bias voltage. In a TEM one also may see that the effective radius can be smaller than expected because there are often small asperities sitting on the large tip close to the surface [22].

The findings in this paper is not only of importance to understand the formation of structures under the STM tip, but may also be of importance in electronics applications for example recently described in ref. [16], where a temporary mound seems to be created between electrodes during a voltage pulse reducing (but not bridging) the gap and thus reducing tunneling resistance several orders of magnitude. Different resistance values can then be interpreted as digital ones and zeroes.

Another application that could be of importance is sintering of nanoparticles for use in printed electronics [35]. The ink used for defining conducting paths consists of nanoparticles that need to be sintered to achieve high enough conductivity. One method is electrical sintering [35], [36] and to model this both the electrical and van der Waals induced surface diffusion models in this paper should be of relevance.

**Figure 7:**
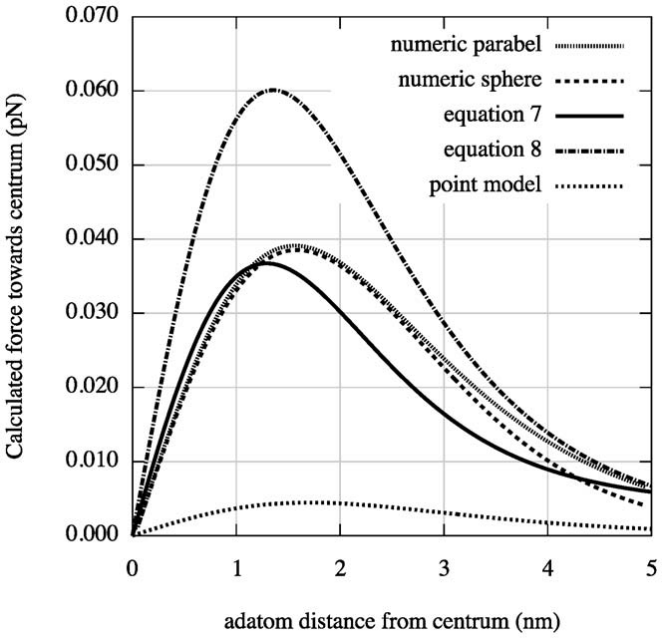
Radial van der Waals force on an adatom from simulations of a parabolic and a spherical tip respectively as a function of the distance 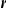 from the centrum under the tip to the adatom using numerical integration of equation (1). Also plotted in the figure is equation (7) and (8). 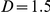 nm and 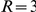 nm. Even the curve of a point model has been added. We see that equation (7) agrees well with the simulated curves close under the tip but underestimates the van der Waals force somewhat for larger 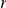 where the approximation of the tip as a sphere becomes too crude. The simpler formula equation (8) assuming 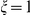 overestimates the force compared with the simulations in the figure. However, even this equation should underestimate the force for even larger 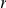 because the assumption of the tip as a sphere that both equation (7) and (8) uses breaks down. That the simulated value for the parabolic tip tend to sink below the curve from equation (8) at 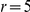 nm is probably due to the limited size of the tip used in the simulation. In deriving equation (5), that equation (7) and (8) uses, we assume that the tip is extended to infinity.

**Figure 8:**
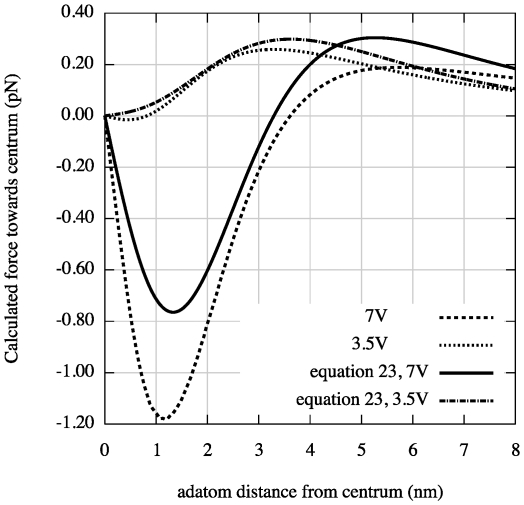
Radial dipole force on an adatom simulated using a conducting parabolic tip over a conducting plane for 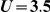 V and 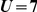 V respectively. Radius of curvature of the tip is 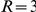 nm and tip-to-surface distance 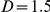 nm. To calculate the electric field 400 positive charges are placed on the tip and 400 negative charges are placed on the surface. They are free to move and do so until they have reached their equilibrium positions. Thereafter the field generated by them is calculated and finally the radial force on the adatom. Also plotted is equation (23) for the same two voltages as above.

We have in this paper considered the static electric- and van der Waals forces on adatoms on a surface under a scanning tunneling microscope tip. We have described an analytical model of this system with three main conclusions: 1) the van der Waals force becomes important for tip-to-surface distances shorter than about 1.5 nm, 2) there is a threshold voltage in mound formation corresponding to a threshold electric field of about 2 V/nm, 3) and there is a relation between applied positive voltage and radius of the mound. To illustrate the result of our analysis we made plots. A plot of 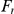 from equation (7) as a function of 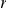 is shown in fig. 4 and fig. 5 and 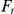 as a function of 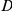 is shown in fig. 6. To verify the van der Waals-model we made simulations. We numerically integrated the van der Waals force given by equation (1) for a parabolic and spherical tip respectively, see fig. 7. In the figure we have also plotted equation (7) and (8). To test the model for the electrostatic dipole force we made a simulation of an adatom on a conducting plane under a conducting parabolic tip. This result is compared to equation (23) in fig. 8.
